# Poorly Differentiated Neuroendocrine Larynx Carcinoma: Clinical Features and miRNAs Signature—A New Goal for Early Diagnosis and Therapy?

**DOI:** 10.3390/jcm10092019

**Published:** 2021-05-08

**Authors:** Filippo Ricciardiello, Michela Falco, Giuseppe Tortoriello, Ferdinando Riccardi, Raul Pellini, Brigida Iorio, Giuseppe Russo, Giuseppe Longo, Ciro Coppola, Takashi Takeuchi, Anna Grimaldi, Marianna Abate, Marianna Scrima, Alessia Maria Cossu, Raffaele Addeo, Alessandro Ottaiano, Alfonso Scarpa, Amedeo Boscaino, Giovanni Motta, Michele Caraglia, Marco Bocchetti, Gabriella Misso

**Affiliations:** 1 U.O.C. Otolaryngology, AORN “Antonio Cardarelli”, 80131 Napoli, Italy; filipporicciardiello@virgilio.it (F.R.); giovanni.motta@aocardarelli.it (G.M.); 2 Department of Precision Medicine, University of Campania “Luigi Vanvitelli”, 80138 Naples, Italy; michela.falco@unicampania.it (M.F.); takashi19900706@gmail.com (T.T.); alessiamaria.cossu@biogem.it (A.M.C.); michele.caraglia@unicampania.it (M.C.); marco.bocchetti@unicampania.it (M.B.); gabriella.misso@unicampania.it (G.M.); 3 U.O.C. Otolaryngology, ASL-NA1 Ospedale del Mare, 80147 Naples, Italy; dott.giuseppetortoriello@virgilio.it; 4 U.O.C. Oncology, AORN “Antonio Cardarelli”, 80131 Naples, Italy; nando.riccardi@gmail.com; 5 U.O.C. Otolaryngology-Head and Neck Surgery, IRCCS Regina Elena National Cancer Institute, 00161 Rome, Italy; pelliniraul@yahoo.it; 6 U.O.C. Otolaryngology, San Paolo Hospital, ASL Napoli 1, 80145 Naples, Italy; brigida.iorio82@gmail.com; 7 U.O.C. General Direction, AORN “Antonio Cardarelli”, 80131 Naples, Italy; giuseppe.russo@aocardarelli.it (G.R.); ciro.coppola@aocardarelli.it (C.C.); 8 U.O.C. Healthcare Direction, AORN “Antonio Cardarelli”, 80131 Naples, Italy; giuseppe.longo@aocardarelli.it; 9 Molecular Diagnostics Division, Wakunaga Pharmaceutical Co., Ltd., Hiroshima 739-1195, Japan; 10 U.P. Diagnostics Cytometric and Mutational, Vanvitelli Hospital, 80138 Naples, Italy; grim.anna@tiscali.it; 11 Laboratory of Molecular and Precision Oncology, Biogem Institute of Molecular Biology and Genetics, 83031 Ariano Irpino, Italy; marianna.scrima@biogem.it; 12 U.O.C Oncology, Day Hospital, San Giovanni di Dio Hospital, ASL Naples 2 Nord, 80020 Frattamaggiore, Italy; raffaeleaddeo19@gmail.com; 13 S.S.D. Innovative Therapies for Abdominal Metastases, Istituto Nazionale Tumori, IRCCS Fondazione G. Pascale, 80131 Naples, Italy; a.ottaiano@istitutotumori.na.it; 14 U.O.C Medicine, Surgery and Dentistry, University of Salerno, 84081 Baronissi, Italy; alfonsoscarpa@yahoo.it; 15 U.O.C Pathologic Anatomy, AORN “Antonio Cardarelli”, 80131 Naples, Italy; amedeo.boscaino@aocardarelli.it

**Keywords:** neuroendocrine, larynx, squamous, poorly differentiated, carcinoma, miRNA

## Abstract

Laryngeal neuroendocrine carcinomas (LNECs) are rare and highly heterogeneous malignancies presenting a wide range of pathological and clinical manifestations. Herein, we retrospectively characterize ten patients diagnosticated with LNEC, five of which were defined as well-moderately differentiated neuroendocrine carcinomas, and five that were defined as poorly differentiated neuroendocrine carcinomas, according to the latest WHO classification. Clinical features were analyzed and compared between the two subgroups together with a microRNA study which evidenced a peculiar signature likely related to poorly differentiated larynx neuroendocrine carcinomas. These findings may offer new useful insights for clinicians to improve diagnosis efficiency, therapy response, and patients’ outcome for this aggressive neoplasm.

## 1. Introduction

Laryngeal neuroendocrine carcinomas (LNECs) are malignant heterogeneous epithelial neoplasms characterized by neuroendocrine differentiation. LNECs are rare, representing approximately 1% of all organ’s neoplasms [1], but are the second most common group of Larynx neoplasms after squamous cell carcinoma [2]. The most conceivable hypothesis is their origin from widespread neuroendocrine system cells [2]. The typical onset is between the 5th and the 7th decade of life and involves the supraglottic region. The symptoms are often related to an obstructive mass lesion, including hoarseness and difficulty in swallowing [3]. Paraneoplastic syndromes have been reported in patients with these tumors as well [3]. The classification of these tumors has evolved throughout the years, and the latest one was described in 2017 in the World Health Organization (WHO) Classification of head and neck tumors [4]. The goal of the current classification is to combine histological characteristics with clinical and prognostic behaviour; neoplasms were differentiated into epithelial and neural. Neural lesions are essentially paragangliomas, which are invariably benign. On the other hand, a grading was associated with the epithelial classification: well-differentiated neuroendocrine carcinoma, moderately differentiated neuroendocrine carcinoma, and poorly differentiated neuroendocrine carcinoma [5]. Ki-67 scoring by immunohistochemistry is also a common method for differential undifferentiated NET diagnosis, as it reflects the percentage of invading undifferentiated cells [5,6,7].

The prognosis is strongly conditioned by grading and subtype of LNECs; consequently, it is important to recognize the WHO classification of head and neck tumors.

miRNAs expression nowadays is crucial in precision and personalized medicine for an early and more accurate diagnosis. These small, non-coding RNAs, approximately 20 nucleotides long, are involved in maintaining homeostasis and exerting post-transcriptional gene expression regulation, mainly by messenger RNA complementary interaction. Moreover, miRNA expression and mechanisms of action on biological pathways appear strictly tissue and cellular context specific, especially in the histological patients’ samples. Their stable form is also present in the bloodstream, even if at low concentrations, often strictly correlating to pathological conditions such as tumors and metastases, pointing out their potential exploitation as early non-invasive biomarkers [8]. We have recently characterized miRNA expression profiles of laryngeal cancer tissues, finding a significant downregulation of miR-133b and miR-449a compared to adjacent normal counterparts. Interestingly, miR-449a also showed a predictive potential for the occurrence of nodal metastases [8]. Moreover, in the validation process, we underlined the diagnostic potential of miR-223, which resulted in significant upregulation in laryngeal tumor samples. A laryngeal cancer-related oncogenic function was also confirmed by Wei et al., which demonstrated the inhibitory effect of hsa_circ_0042666 on proliferation and invasion through the targeting of miR-223/TGFBR3 axis [9,10,11]. Based on these latest data, we focused on these three miRNAs to investigate their modulation also in LNECs.

Therefore, the aim of this study is to report our experience on a series of patients with LNECs to identify clinical features, treatment outcome, follow-up, overall survival, and miRNAs profiling to propose a diagnostic-therapeutic signature.

## 2. Materials and Methods

### 2.1. Tissues and Patients

Patient cohort was selected among patients of the Ear-Nose-Throat (ENT) Unit at Cardarelli Hospital, Naples (Italy); ASL Napoli 1 Hospitals, Naples (Italy); and from “IFO”, Rome (Italy). Patients were selected during 1 January 2002 to 31 October 2019. Patients enrolled in the study underwent ENT examination by laryngoscopy, neck, and chest CT with intravenous contrast, biopsy in direct microlaryngoscopy (MLDS), followed by histological examination that confirmed LNEC diagnosis. We collected patients’ data: age, gender, tumor site, preoperative imaging, treatments, outcome, endoscopic and radiological follow-up (Table 1). Patients with LNEC were treated with cordectomy performed with CO_2_ laser aid or open laryngectomy (subtotal or total). Laterocervical lymph node dissection (LLND), prophylactic or curative, was performed in 9 patients, followed, after oncological specialist evaluation, by adjuvant chemoradiotherapy in 7 cases. In the post-operative time, patients underwent oncological follow-up, examinations by ENT specialist with direct fiber optic laryngoscopy and video recording in accordance with the timetable guidelines for each tumor stage, annual neck and chest CT, and total body PET for both loco-regional and distance disease recurrence monitoring. pTNM was evaluated according to TNM criteria (VIII edition) [12]. Patients with follow-up lower than 18 months were excluded from the study, this being the minimum time for an accurate evaluation of survival. Based on this premise, only one patient with a 7-month follow-up was excluded. The demographic characteristics (staging, grading treatment strategies, and radiotherapy) for each patient subgroup are described in Table 1. According to these inclusion criteria, the study population was formed by 10 consecutive cases of LNEC (7 M and 3 F). Moreover, 75 LCa tissues were taken from patients suffering from Larynx squamous cell cancer (SCC). Normal Adjacent Tissue was also collected for each patient. Tissue specimens were introduced into RNAlater (Ambion, Life Technologies, Carlsbad, CA, USA) and kept at 4 °C until RNA extraction. The study was in accordance with the Institutional Ethics Committee guidelines, Italian law, and the Declaration of Helsinki, as required for studies based on retrospective analyses on routine archival formalin-fixed, paraffin-embedded tissues. All patients provided written informed consent regarding the use of these data for research purposes.

### 2.2. RNA Extraction

Total RNA, including miRNA, was extracted from approximately 50 mg of clinical tissue specimens using a mirVana PARIS kit (Ambion, Life Technologies, Carlsbad, CA, USA) according to the manufacturer’s protocol with the following modification: RNA was finally eluted into 50 µL of pre-heated elution buffer in order to concentrate the extract. RNA purity and quantity were measured by a spectrophotometer using the 260/280 nm ratio with a NanoDrop ND-1000 (Thermo Scientific, Wilmington, NC, USA). RNA samples were stored at −80 °C until further processing.

### 2.3. RT-qPCR for miRNA and mRNA Expression

miRNA expression was evaluated on a total of 75 SCC tissues, 5 poorly differentiated NET and 5 differentiated NET compared to the respective normal adjacent tissues.

cDNA synthesis was performed using a TaqMan miRNA reverse transcription kit (Applied Biosystems, Foster City, CA, USA) for three miRNA candidates (miR-133b, miR-449a, and miR-223) in accordance with the manufacturers’ instructions. The expression levels of miRNA candidates were detected using TaqMan Fast universal PCR master mix (Applied Biosystems, CA, USA) via the ViiA 7 real-time PCR system (Applied Biosystems, Foster City, CA, USA) with each primer for TaqMan miRNA primers (Applied Biosystems, Foster City, CA, USA).

Real-time PCR was performed on a ViiA 7 real-time PCR system. The Ct value of each miRNA was determined using ViiA 7 software (Applied Biosystems, Foster City, CA, USA) and setting a threshold of 0.2 Ct values. For miRNAs undetermined by the instrument the threshold was set up at 35.0. For calculating the ∆Ct of miRNAs of interest, Ct values of each miRNA were normalized with U6 small nuclear RNA (snRNA) as an endogenous control, and mean Ct values of a miRNA across five pools were used. The relative miRNA expression was calculated with the ∆∆Ct method. FC was calculated using the 2 ^−∆∆Ct^ method [10]. Each sample was run in triplicate.

### 2.4. Statistical Analysis 

Survival analysis was performed using the Kaplan-Meier method, normalizing the different categories by the long-rank Mantel-Haenszel test, reported as mean with 95% confidence intervals (CI) using Med-Calc software, version 9.3.7.0. We applied the Kaplan-Meier method in order to study overall and disease-specific survival. Overall survival (OS) is defined as the time from first diagnosis to death from any cause or the date of the last follow-up. Disease specific survival (DSS) is defined as the time from diagnosis to death for the disease. In each test, *p* value < 0.05 was considered statistically significant. Wilcoxon/Mann-Whitney test was used for independent and non-parametric variables.

Pearson correlation and related graphs were generated using IBM SPSS software (SPSS software, Ver. 25, IBM Corp., Armonk, NY, USA), while GraphPad PRISM (GraphPad PRISM software, Version 8, San Diego, CA, USA) was used for the miRNAs expression graphs.

## 3. Results

### 3.1. Demographic and Clinical Characteristics of the Patients

The study cohort was formed by 10 cases of LNEC (7 M and 3 F). The clinical features are summarized in Table 1. Seven patients were males and three females. The median age at the time of diagnosis was 63.1 years (range 49–81). Voluptuous habits investigated were smoke and alcohol; eight (80%) patients were smokers and five (50%) smokers and alcohol users. At the time of diagnosis, most patients, nine (90%), showed neoplasm localization at the supraglottic region; in details, five cases (50%) showed exclusively supraglottic neoplasm and four showed both supraglottic and glottic involvement (40%); the remaining case showed hypopharyngolaryngeal localization. According to TNM classification VIII edition, eight patients (80%) were in stage IV, one (10%) in stage III with a liver synchronous localization and one (10%) in stage I; each pTNM was showed in Table 1. On the other hand, histological grading was poorly differentiated neuroendocrine carcinoma in five (50%) patients and, among these, three large cell cancer and two small cell cancer patients; moderately differentiated neuroendocrine carcinoma (G2) was found in three patients (30%) and well-differentiated (G1) in two cases (20%).

### 3.2. Patient Management

After biopsy in MLDS for histological diagnosis, all patients were surgically treated; enlarged laser supraglottic cordectomy and horizontal partial laryngectomy (OPHL I) were performed in one (10%) and three patients (30%), respectively. Five patients (50%) were treated with total laryngectomy (TL) and one patient (10%) with hypopharyngolaryngectomy. In nine patients (90%), prophylactic or curative LLND was performed. Adjuvant treatments were administered in seven patients (70%), exclusive radiotherapy (RT) in three cases (30%), combined chemoradiotherapy in three cases (30%), and chemotherapy in one patient for hepatic disease control.

### 3.3. Clinical Statistical Analysis

No T recurrence was reported, but one (10%) N recurrence and five (50%) distant metastases were recorded. Four patients (40%) are alive without clinical and radiological signs of disease recovery; two (20%) are alive in chemoradiotherapy metastatic disease control; four patients died (40%), three from the disease (30%) and 1 from other reasons.

Analyzing odd ratio (OR) mortality risk between the poorly differentiated and the well-moderately differentiated subgroups, we found an increased risk in the first one of 2.6667 (95% CI = 0.1575 to 45.1435; z statistic = 0.680; *p* value = 0.4968) with a relative risk of 1.333 (95% CI = 0.5761 to 3.0861; z statistic = 0.672; *p* value = 0.5017). Despite the small number of cases the difference in aggressiveness between the poorly differentiated and the well-moderately differentiated subgroups is likely attributable to distant metastases. Applying this feature to calculated OR between the poorly differentiated and the well-moderately differentiated subgroups, we found a 16-fold increased distant metastases risk (95% CI = 0.7215 to 354.8236; z statistic = 1.754; *p* value = 0.0795). On the other hand, the relative risk of distant metastases is 4-fold less in the poorly differentiated subgroup (95% CI = 0.6566 to 24.3693; z statistic = 1.504; *p* value = 0.1327).

**Figure 1:**
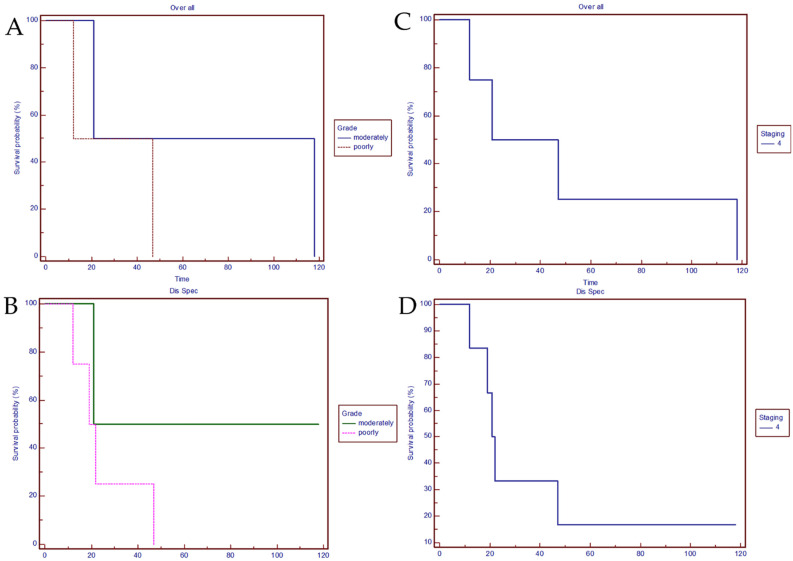
(**A**) Overall survival. Comparison of the poorly differentiated subgroup and the moderately differentiated subgroup. (Chi-square 0.6154 DF 1; *p* = 0.4328; hazard ratio 0.5000 95% CI 0.03958 to 3.878). (**B**) Disease specific survival. Correlation between the poorly differentiated subgroup vs. the moderately differentiated subgroup. (Chi-square 0.9606 DF 1; *p* = 0.3270; hazard ratio 0.3548 95% CI 0.0671 to 2.4605). (**C**) Overall survival correlated to staging. (**D**) Disease specific survival correlated to staging.

The analysis for overall survival (OS) evidenced 29.5 months median survival in the poorly differentiated subgroup and 69.5 months in the well-moderately differentiated subgroup (Figure 1A). The 18-month survival rate was 80% in the poorly differentiated subgroup and 100% in the well-moderately differentiated subgroup.

Considering specific disease survival (DSS), including recurrence as negative endpoint, any patient in the well-moderately differentiated subgroup died from disease; median survival in the poorly differentiated subgroup was 20.2 months (Figure 1B).

Analyzing overall survival correlated to staging, median survival was 34.0 months for stage IV (Figure 1C).

Analyzing disease specific survival correlated to staging, median survival was 21.5 months for stage IV (Figure 1D).

Poorly differentiated LNEC positively correlates with nodal metastasis (N+) and relapse as showed by the significative Pearson correlation (Table 2).

**Figure 2:**
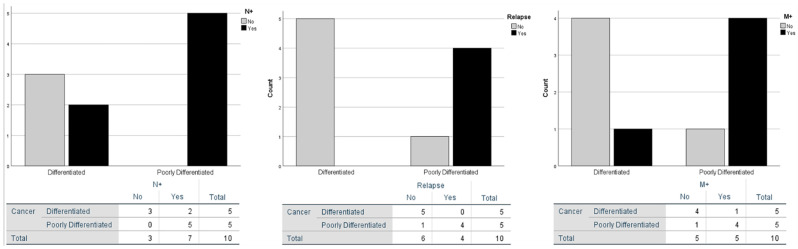
Frequency of N+, M+ and relapses. Comparative relationship between poorly differentiated and differentiated neuroendocrine carcinoma of the larynx.

The frequency tables report the above-mentioned significant correlation and bar chart graphs, including metastases, which appear impactful as well (Figure 2).

Those features underline the significant clinical differences reflecting the poorly differentiated LNEC aggressiveness.

### 3.4. MicroRNA Analysis

**Figure 3:**
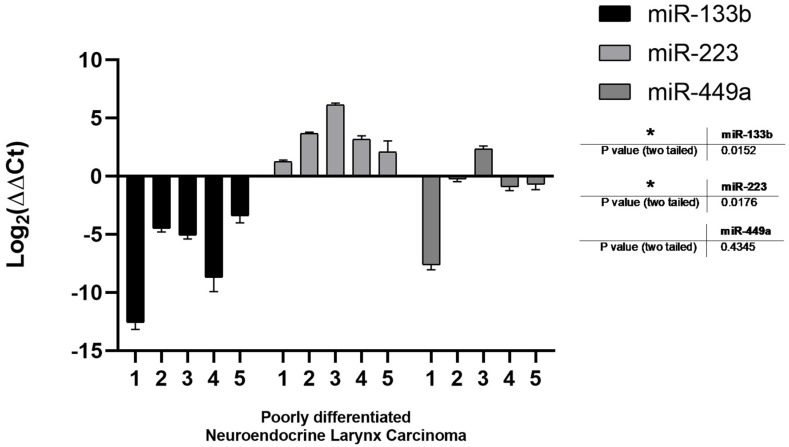
MicroRNA analysis. Different miRNAs expression in the poorly differentiated neuroendocrine larynx tumors compared to normal adjacent tissue (and differentiated neuroendocrine larynx tumors). *p* values from one sample *t* test analysis. * Statistically significant difference: *p* < 0.05.

Thereafter, we performed a quantitative RT-PCR to assess the expression of the following three miRNAs of interest in laryngeal cancer: miR-133b, miR-223 and miR-449a. Of note, our results enlighten a precise expression pattern in poorly differentiated LNEC compared to normal adjacent tissue. In details, in all of our specimens, miR-133b was remarkably downregulated while miR-223 had an opposite trend. miR-449a was also downregulated in 4 out of 5 poorly differentiated patients; however, overall, its modulation does not appear to change significantly (Figure 3). Moreover, well differentiated and moderately differentiated LNEC does not show a significant modulation for each of the three miRNAs compared to normal adjacent tissues.

**Figure 4:**
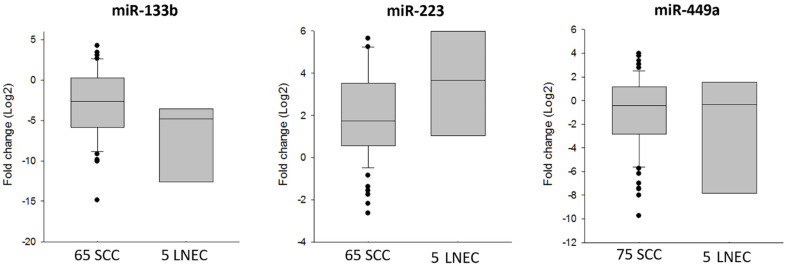
MicroRNA analysis. Different miR-133b, miR-223 and miR-449a expression levels in SCC compared to LNEC.

These three miRNAs were also evaluated in a total of 75 larynx SCC patients (65 for miR-133b and miR-223 and 75 for miR-449a). The same trend was observed comparing the SCC Log2 (FoldChange) to the poorly differentiated LNEC as shown in the graph below (Figure 4). The results suggest a more marked miR-133b down-regulation and miR-223 up-regulation in LNEC, while miR-449a expression was similar in both LNEC and SCC. However, additional validation on a larger cohort is warranted to confirm the significance of the modulation herein reported.

These findings suggest that this miRNA signature should be a peculiar characteristic of poorly differentiated LNEC, providing the rationale for a subsequent validation on a larger cohort.

## 4. Discussion

### 4.1. Clinical Evidence

In 2017, WHO clarified neuroendocrine neoplasms’ nomenclature to unify the different definitions found in the literature during the previous years. LNECs are rare, aggressive and malignant group of tumors with different prognosis, characterized by neuroendocrine cells’ differentiation. Neuroendocrine carcinoma derives probably from the cells of the widespread neuroendocrine system.

The retrospective study by Zhu et al. reported a mean survival time of 112.5 months (95% CI, 81.5–143.6) for 14 LNEC patients classified into three subtypes: typical carcinoid, atypical carcinoid, and small cell neuroendocrine carcinoma. The 2-year and 5-year survival rate in these series were 84.4% and 73.9%, respectively. However, the authors concluded that each LNEC subtype requires a different treatment protocol and shows a peculiar clinical behavior and prognosis [13].

The prognosis of small cell neuroendocrine carcinoma of larynx was very poor with 5-year survival rates less than 10% [14,15,16]. However, Zhu et al. reported for these patients a mean survival time of 79.7 months (95% CI, 37.9–121.4) with a 5-year survival rate of 53.6%, which was much higher than previously reported in literature [13].

In our study, we included: (a) five patients (50%) with poorly differentiated neuroendocrine carcinoma, including three cases with large cell and two with small cell carcinoma; (b) three patients (30%) with moderately differentiated (G2) neuroendocrine carcinoma; (c) two (20%) with well-differentiated (G1) neuroendocrine carcinoma. We observed a very different clinical and prognostic behaviour between poorly differentiated neuroendocrine carcinoma and well-moderately differentiated (G1 and G2) neuroendocrine carcinoma. G1 and G2 carcinomas behave similarly to SCC, showing a median survival of 69.5 months, while in the poorly differentiated subgroup median survival dramatically decreases to 29.5 months, demonstrating increased aggressiveness, especially due to the risk of distant metastases.

Mortality risk, described by OR, showed a 2.6667-fold increased risk in poorly differentiated subgroup correlated with grading. The added risk (OR) of developing distant metastases was 16-fold higher in poorly differentiated patient subgroup. Therefore, it should be considered a careful timing of total body radiological investigations for these patients and, certainly, not the same as for patients with SCC or well-differentiated neuroendocrine cancers. Our follow up protocol in case of poorly differentiated LNECs includes:

- fibroscopy of the upper respiratory tract every month for three months, every three months for two years, and then every six months for three years;
- total body TAC-PET six months after the end of the treatment and then every six months for two years.

Despite the low number of cases reported, the present study offers the possibility of broadening the current knowledge relevant to the clinical and molecular characterization of a very rare neoplasia, which currently includes just about 500 total cases throughout the literature. Moreover, to our knowledge, this is the first study analyzing miRNA expression profile in LNEC.

### 4.2. miRNAs Clinical Value

miR-133b is generally considered an oncosuppressor. Several studies demonstrate its oncosuppressive role in laryngeal cancer, where it showed a significantly lower expression in both serum [17] and tissues of patients [8], compared to healthy individuals and surrounding normal tissue, respectively. It is generally downregulated in cancerous tissues [18] and its low expression is considered as a biomarker for cancer migration and invasion [19], but the underlying mechanisms are still not clear. miR-133b overexpression via transfection reverts the aggressive phenotype even in renal carcinoma [20]. Moreover, it was reported to play a role in resistance to chemotherapy and, generally, in response to therapy. In fact, miR-133 upregulation increases the cisplatin chemosensitivity of cisplatin-resistant non small cell lung cancer (NSCLC) cells. Together with TUG1 and CRC4, it is involved in cisplatin resistance even in tongue squamous carcinoma patients [21,22]. Similarly, the findings herein reported, suggest a possible onsuppressive role for miR-133b due to its prominent downregulation in LNEC tissues.

miR-223 was found upregulated in both tissues and plasma of head and neck squamous cell carcinoma (HNSCC) patients. Interestingly, miR-223 levels decreased after surgery, except in the case of cancer recurrence, thus suggesting a possible prognostic role for this miRNA [23]. Increased levels of miR-223 were observed even in pediatric lymphoblastic T cell lymphoma where it represents an independent worse prognosis factor [24]. In NSCLC patients’ saliva, miR-223 levels were also upregulated compared to healthy controls [25] and the same trend was also observed in gastric and esophageal cancers patient tissues [26]. Moreover, miR-223 expression was associated to platinum-based compounds chemotherapy resistance in triple-negative breast cancer and was correlated to the inhibition of resistance to doxorubicin-induced autophagy and to chemotherapy in hepatocellular carcinoma cells [27,28]. Consistent with previous findings, the present study also delineates a marked up-modulation trend for miR-223, thus suggesting also in this case an oncogenic function.

miR-449a is generally studied as a tumor suppressor, as its overexpression is mostly protective [29], and it is downregulated in various cancerous tissues. It was associated with cell senescence and inhibited cancer cell migration, invasion, and growth [29,30], underlying its potential application in cancer diagnosis and therapy. In gastric cancer, it was recognized as a prognostic predictive biomarker [31]. Moreover, it was correlated with enhanced response to radiotherapy in prostate cancer [32]. We have previously reported that its overexpression in laryngeal cancer tissues was associated with a lower risk to develop regional lymph node metastases and that it reduced invasion and proliferation of laryngeal cancer cells in vitro likely through the dysregulation of NOTCH-1-dependent pathway [8]. Of note, in the present study, we have observed for miR-449a an uneven trend that produces an overall modulation similar to larynx SCC. 

However, a subsequent expansion of LNEC patients enrolled in the study will be required in the near future to assign a statistical significance to our data.

The specific miRNA signature herein described for LNEC could be not only useful for diagnostic purposes, but also to develop new treatment strategies, given their present limitation due to the poor results derived from the administration of the conventionally approved treatment. In fact, one of the most important limits in the definition of the best therapeutic strategies in LNEC is due both mainly to the retrospective nature of the studies and to the existence of just few non-controlled small trials [13,33].

Patients are often treated similarly to the more common small cell lung carcinoma (SCLC). These studies have a larger number of patients enrolled and act as a guideline for the other poorly differentiated NECs of the other districts. For 25 years, cytotoxic treatment with cisplatin and etoposide has been used as standard treatment for metastatic NEC. Moertel and colleagues described an overall remission rate of 67% and a median survival of 19 months [34]. Response to treatment was independent from primary tumor localization. Mitry et al. reported comparable results with an RR of 42% and a median survival of 15 months using a modified cisplatin and etoposide regimen in a cohort of 41 patients [35].

In the scheme for the first line, cisplatin can be performed on day one at 100 mg/m^2^ or divided into days 1–2 at 50 mg/m^2^, then etoposide at 100 mg/m^2^ on days 1–3 [36]. The cycle can be repeated every three weeks. However, the prognosis remains poor with a two-year survival lower than 20%, confirming that new therapeutic strategies have to be developed.

The three deregulated microRNAs in our poorly differentiated LNEC subset of patients, represent a clear signature offering a neat overview on the malignancy and aggressiveness of the tumor evidenced and confirmed by clinical evaluation as well. Moreover, these miRNAs might concretely be involved in chemotherapy failure especially with cisplatin.

## 5. Conclusions

MicroRNAs are becoming more and more important given their crucial role in both clinic and translational medicine. They represent a novel approach to diagnosis, exploited as early non-invasive and potentially predictive biomarkers of response to chemotherapy. Regarding their therapeutic potential, precision medicine tied on peculiar and specific patient profile has recently focused on the excellent opportunities arising from the possibility of restoring homeostatic miRNA levels. LNECs are the most common types of non-squamous neoplasms of the larynx. Owing to their heterogeneity, clinical behaviors, treatment protocols, and prognosis are different for each subtype, particularly between well-moderately differentiated carcinomas and poorly differentiated ones.

Therefore, a thorough characterization for each type of neuroendocrine neoplasms of larynx is necessary for both pathologists and clinicians in order to appropriately treat these aggressive diseases. Clinical features, coupled with novel, accurate, and specific differentiation biomarkers, such as microRNAs, might be a crucial and powerful weapon to be exploited to improve diagnosis, specific therapy, and, consequently, patient outcome.

## Figures and Tables

**Table 1:** Patient characteristics. The table provides an overview of patient characteristics including gender, age, potential influencing factors, tumor site, histological classification and therapeutic treatment of the malignancy.

Patient	1	2	3	4	5	6	7	8	9	10
**Age**	69	58	55	58	60	70	49	57	81	74
**Sex**	M	F	M	F	F	M	M	M	M	M
**Voluptuous Habits**	Smoke	Smoke Alcohol	Smoke	None	Smoke	None	Smoke Alcohol	Smoke Alcohol	Smoke Alcohol	Smoke Alcohol
**Tumor site**	Supraglottic/Glottic	Supraglottic	Glottic/Supraglottic	Supraglottic	Supraglottic	Supraglottic	Hypopharyngolaryngeal	Glottic/Supraglottic	Glottic/Supraglottic	Supraglottic
**Histological Differentiation**	Poorly Large Cells	Poorly Small Cells	Poorly Large Cells	Poorly Small Cells	Moderately G2	Well G1	Moderately G2	Well G1	Poorly Large Cells	Moderately G2
**Diagnosis**	Nov 15	Sep 17	Jan 18	Feb 18	Jan 18	Feb 17	Feb 18	Feb 17	Dec 14	Dec 09
**Initial Treatment**	TL	TL	TL	OPHLI	OPHLI	Laser Cordectomy	Hypopharyngolaryngectomy	TL	TL	OPHL I
**pTNM**	PT4aN3M0	PT4aN2aM0	PT3N3AM0	PT3N2AM0	PT3N2BM0	PT1N0M0	PT4ANOM0	PT3N0M1	PT4aN3M0	PT4aN2aM0
**Neck Dissection**	LLND	LLND	LLND	LLND	LLND	NO	LLND	LLND	LLND	LLND
**Adjuvant Therapy**	ChRT	RT	ChRT	ChRT	RT	NONE	RT	Ch	NONE	NONE
**Follow Up (months)**	47	25	22	19	22	30	21	30	12	118
**Vital Status**	† M	Follow Up	M	M	Follow Up	Follow Up	† M	Follow Up	† N Recurrence And M	† Other Cause

Tl: total laryngectomy; Ophl I: horizontal partial laryngectomy type I; LLND: laterocervical lymph node dissection; Ch: chemotherapy; RT: radiotherapy; †: death; M: metastasis; N: lymph node metastasis.

**Table 2:** Pearson correlation. Table shows the association between poorly differentiated neuroendocrine carcinoma of the larynx and distant metastases (M), nodal metastases (N+), or recurrence.

		Poorly Differentiated LNEC	M+	N+	Relapse
Poorly differentiated LNEC	Pearson Correlation	1	0.600	0.655 *	0.816 **
	Sig. (2-tailed)		0.067	0.040	0.004
	*N*	10	10	10	10
M+	Pearson Correlation	0.600	1	0.218	0.816 **
	Sig. (2-tailed)	0.067		0.545	0.004
	*N*	10	10	10	10
N+	Pearson Correlation	0.655 *	0.218	1	0.535
	Sig. (2-tailed)	0.040	0.545		0.111
	*N*	10	10	10	10
Relapse	Pearson Correlation	0.816 **	0.816 **	0.535	1
	Sig. (2-tailed)	0.004	0.004	0.111	
	*N*	10	10	10	10

* Correlation is significant at the 0.05 level (2-tailed). ** Correlation is significant at the 0.01 level (2-tailed).

## Data Availability

Not applicable.
